# Managing the end of life in COVID patients. The role of palliative care in emergency departments during the pandemic

**DOI:** 10.3389/fsoc.2022.1039003

**Published:** 2022-11-09

**Authors:** Barbara Sena, Enrico De Luca

**Affiliations:** ^1^Department of Law and Economics, Unitelma Sapienza University, Rome, Italy; ^2^Medical School, University of Exeter, Exeter, United Kingdom

**Keywords:** COVID-19, end-of-life care, palliative care, emergency departments, interprofessional care

## Abstract

Managing COVID-19 patients has been an extremely difficult and dramatic task, especially for emergency departments during the strongest waves of the pandemic in Italy. Medical staff and health professionals were redeployed from their work setting to COVID units; many were overwhelmed by the deaths of so many patients in a very short time. This work aimed to explore palliative care health professionals' and physicians' perceptions of end-of-life care management in COVID units during the first two waves of the pandemic in Italy. Qualitative data was collected through 24 semi-structured in-depth interviews. The participants were palliative care medical and health professionals redeployed, or in a supporting role, COVID units from the most affected areas of northern and central Italy. The interview questions were focused on four thematic areas concerning different aspects of the role and responsibilities of the palliative care specialist (physician and healthcare professional). A brief presentation of the main sociological literature on end-of-life management in hospital contexts will be firstly presented and discussed to offer a theoretical frame. Subsequently, some of the most significant results that emerged from our research will be illustrated concerning the role played by palliative care professionals during the pandemic and the relevance of the palliative care approach in emergency contexts.

## Introduction

In the most critical phases of the COVID-19 pandemic, the management of the end of life has been an extreme difficult task, especially in the emergency and intensive care departments. The high number of patients affected by COVID-19 (from now on only COVID) quickly saturated these wards, which had very few resources available to cope with this new emergency. Many doctors and health professionals were suddenly forced to come out of their daily routine and they have been overwhelmed by the deaths of so many patients, and it has been reported, in some cases, severe psychological, occupational and physical consequences (Leo et al., 2021; Silverman et al., 2021; Søvold et al., 2021).

In Italy, especially during the first wave of pandemic, with its rapid increase in COVID cases and deaths, mostly in the north of the country, healthcare personnel often found themselves managing the end of life of COVID patients alone. Relatives and caregivers were not allowed to stay, assist, and often, even to see their beloved once admitted in the hospital. In the most critical period of the pandemic, the increasing difficult situation of many COVID wards, and their limited resources and capacity, has even led the Italian Society of Anaesthesia (SIAARTI, 2020) to issue emergency guidelines for doctors. These guidelines recommended doctors to reserve intensive care or eventual escalation of treatments (such as endo tracheal intubation, invasive ventilation, etc.) only for patients who could have a higher probability of “therapeutic success”. The decision was solely based on doctors' clinical judgement without any involvement from other professionals and the family members, therefore without the wider consensus generally expected in any end of life decision.

However, the second wave of infections also saw strong pressure on hospitals in various Italian regions, recording an even higher mortality rate of COVID patients than the first (Istat ISS, 2021).

In this situation, palliative care teams provided support in many COVID wards, trying not only to manage the end of life of dying patients, but also to help colleagues by providing them with professional and psychological support (Singh et al., 2021).

Humanitarian crises and health emergencies, events generally characterized by high mortality rates, have excluded the involvement of palliative care (Nouvet et al., 2018). This paradox, however, has been highlighted recently in literature, pointing out the importance of including palliative care also in these cases (Wynne et al., 2020). In 2018, the WHO also formulated a guide on the topic, emphasizing the need to integrate palliative care in contexts where the humanization of care and patient treatment often tends to fail due to scarcity of resources, high number of deaths and the inability of health services and health professionals to provide adequate care to save the patient (WHO, 2018). Although palliative care teams showed to possess consistent skills and training for responding to health emergencies, there are still not many investigations on their real contribution in emergency departments and intensive care units, particularly during the most critical phases of the COVID pandemic.

Starting from this premise, this work aims to explore the experience and point of view of health professionals and physicians specialized in palliative care, who were deployed (or reported to have played a supportive role) in emergency departments during COVID pandemic in Italy. Specifically, this work will aim to have palliative care team perspectives on the management of end-of-life care in the emergency department.

A brief presentation of the main sociological literature on end-of-life management in hospital contexts will be firstly presented and discussed to offer a theoretical frame. Subsequently, some of the most significant results that emerged from our research will be illustrated in relation to the role played by palliative care professionals during the pandemic and their perception of the relevance of the palliative care approach in emergency contexts.

## Sociological contributions to the management of the end of life in hospital contexts

Interdisciplinary research and, in particular, sociological research, have considered a series of questions around the end of life of a patient and the health organization of death and dying.

First studies, in the early 60s, dealt with the orchestration of dying within organizational structures in hospital settings (e.g., Glaser and Strauss, 1965, 1968; Sudnow, 1967).

In *Awareness of Dying*, Glaser and Strauss (1965) observed how in the hospital setting US doctors were reluctant to reveal impending death to their patients, while nurses were not authorized to give information about it without the doctors' consent. This was due to a generalized attitude in the American culture of the time, which did not allow speaking openly about death, even when this event was personal. The behavior of the hospital staff, analyzed by Glaser and Strauss, was defined by the authors as characterized by different levels of awareness of dying: a *close awareness*, a *suspicion awareness*, a *mutual pretense of awareness* and an *open awareness*. The prevalence in hospitals observed by Glaser and Strauss was to maintain a close awareness of the patient, which meant to deny him knowledge of his true state of dying. At the most, there was an awareness of reciprocal pretense between doctor and patient on his actual end-of-life conditions but, less frequently, there was an *open awareness*, through a mutually and explicitly recognized imminence of death.

Although this research was set in the hospital context of the 1960s in the US, it is still considered very topical. In fact, in the most recent practice we can observe how nurses and doctors continue to check the information given to the patient about his dying state, by delaying, modifying or avoiding the moment of open awareness as much as possible (Andrews and Nathaniel, 2015). Therefore, Glaser and Strauss findings are still very relevant today and continue to highlight problematic aspects of care in health organizations, especially in the contexts of intensive care units (Bandini, 2020).

In the same period, Sudnow (1967), in the ethnomethodological study “*passing on”*, observed that medical and nursing staff organized a set of practices and routine interactions resulting in the so-called patient's “social death”. This situation consisted in healthcare staff considering a dying patient as essentially already a corpse, although still biologically and clinically alive; therefore, the person wasn't involved in his/her care and very limited interactions were planned or happened. Such a situation also influences and determines the medical choices regarding resuscitation attempts to be adopted on the patient.

In the following years, sociologists interested in death and dying in healthcare settings continued their research on several aspects like the ethical issues of medicine and intensive care (Timmermans, 1999), the end-of-life decision-making process (Anspach, 1993; Jenkins, 2015; Bandini, 2020), the medical management of death (Kaufman, 2005; Timmermans, 2005) and the non-ordinary treatments for dying patients (Kaufman, 2015).

Kaufman's (2005) anthropological work on the time of death in hospitals, for example, provides a complete picture of the complexity surrounding decision-making on end-of-life care. In his more recent work, Kaufman (2015) focuses on the boundaries between too many and too few treatments and on the healthcare system' ethical, cultural and political issues that make it difficult to understand such boundaries. His concept of “ordinary” medicine explains how new and advanced screening and treatments become normal in current medicine and how these represent a “standard of care” around which doctors determine what is considered as an excessive or insufficient treatment for the patient care (Kaufman, 2015).

Timmermans (2005), on the other hand, notes that the medical profession can exercise a sort of professional authority in dealing with certain types of death. In an era of advanced therapies and medical technologies, there are different levels of alternative “good deaths” that doctors use to propose family members when they have to decide about the end of life of their relative. However, although technological advances help in saving the lives of critically ill patients, have reinforced dilemmas over decisions regarding end-of-life management, recovery and resuscitation interventions, because they lead physicians to offer patients and their families different options about possible treatment through an increasing shared decision-making process (Seymour, 2001). This kind of behavior, though, has produced as a result an attempt to “normalize” and make end-of-life management as non-conflictual as possible, especially in the hospital setting. In fact, advanced treatments, which were once considered new and not very widespread (such as intubation, oxygenation of extra corporal membranes or dialysis in intensive care), have become ordinary medical practices, constituting a sort of “buffet of choices” or “à la carte menu” submitted to family members, especially when doctors find difficult to decide on the type of treatment to be reserved for their dying relative (Bandini, 2020). This has progressively shifted the “onus” of the end-of-life decision from solely the medical profession into a shared decision making process. However, according to some authors, the metaphor of the “buffet of choices” can reduce burn-out and moral distress experienced by healthcare professionals in the contexts of patients in dying conditions (Hamric and Blackhall, 2007). In fact, several studies have highlighted a variety of situations that can generate ethical and moral conflicts in the management of the dying patient, especially in intensive care and emergency units (Fossum-Taylor et al., 2020). Other authors highlighted problems of communication and disagreement between doctors and nurses over decisions to maintain or discontinue life support treatments for dying patients or the lack of time to provide quality care (Ferrand et al., 2003).

One of the prevailing explanations on the difficulty of managing the process of accompanying death in many clinical contexts is linked to the fact that the final result of end-of-life care is “by definition” the patient's death, that is, according to the principles of medicine, essentially perceived as the “failure” of the cure by clinicians (Bishop, 2011; Gawande, 2014). This conception derives in part from the “clinical mentality” mentioned by Freidson (1988) which consists of an active and pragmatic medical orientation, aimed at making immediate decisions that can always allow a concrete improvement of the health conditions of the patient or a continuous recovery of his vital functions. This mentality implies to put aside any sort of scepticisms, fears and uncertainties about the possibility that the patient may not survive. When such decisions are no longer possible, because medicine is not able any more to cure and heal the patient, different strategies begin to take over to get away from the “problem” of having to manage the end of life; thus, medical profession perceived this instance as cessation or abandonment of power over the patient.

This process, therefore, highlights the cultural authority of medical professionalism, which tries to distinguish the line between to cure and to let die, pursuing the “good death” for the patient and avoiding bad deaths, determining the lifestyles to be promoted and therapeutic changes to make the patient survive as long as possible. In this sense, the end-of-life management can be seen as a professional result (Abbott, 1988) that is subject to incursions from *competitors*, such as palliative care specialists, and to redefinitions of different domains established by the involved health professionals. Not surprisingly, the role of the palliativist professionals still seems to be poorly considered and often subjected to a series of prejudices, not yet demystified by literature and clinical practice. This is due in part to the medical treatment of end-of-life, which implies palliative care for the dying patient, and therefore, it is only initiated when medicine can no longer intervene to save him/her (Masel and Kreye, 2018; Shen and Wellman, 2019). On the contrary, at the basis of the palliative approach there is not only the value of life, which must be lived in the best possible way until the end, but also the value of death, considered as a natural and inevitable event and which, therefore, must not be accelerated, nor postponed unnecessarily, but accompanied to the end.

Palliative care management is a very complex practice in healthcare services; it involves different professionals, ethical and moral choices, minimizing pain, maintaining the dignity of the dying person, facilitating patient and their relative's preferences. For these reasons, palliative care requires very specific and specially trained medical-health figures, who are able to apply a holistic approach, for the best interest and the good of the patient, which includes knowledge and experience in non-traditional areas of medicine (Aldridge et al., 2016). Therefore, a palliative model of care will unequivocally strive (more than other approaches) to provide person centered care and support optimal end-of-life management (Schofield et al., 2021). Having said that, palliative care professionals are strongly suggesting that end-of-life care must be a broad activity in which all medical specialties and professions should be involved at any level (Masel and Kreye, 2018). Thus, an important palliative care team role is related to encouraging and educating other health professionals to manage end-of-life care effectively with an integrated perspective (Kim, 2020).

## The results of the research

### Method

The goal of this research is to explore the perspective and experience of palliative care professionals during pandemic and their role in supporting doctors and nurses in COVID wards.

The study was conducted through a qualitative study design aimed at examining the perspective and experience of different professionals specialized in palliative care, who were deployed and worked in COVID wards during the first two waves of the pandemic.

Qualitative data was collected through 24 semi-structured in-depth interviews. The interview's questions were focused on four thematic areas concerning different aspects of the role and functions of the palliative care specialist (physician and healthcare professional); thus, trying to capture and analyse participants' experience during critical phases of the pandemic.

The interviews were carried out online (through zoom or meet) and recorded, and they were conducted in two different periods: after the first wave of COVID infection (May–July 2020) and after the second wave (May–July 2021).

The participants, a convenience sample, were recruited through personal contact and networks, like the Italian palliative care society (SICP) and two post-graduate courses in palliative care for health professionals (see Table 1).

**Table 1:** Participants' characteristics.

Age: mean (SD)	47.04 years (11.9)
Gender: female *n* (%)	19 (79.1)
**Professional experience in PC:**
Mean (SD)	15.45 years (10.1)
Range	2–35 years
**Healthcare setting/sector (%)**
Hospice	(29.2)
Community services	(29.2)
PC units and/or PC team actively working in hospitals	(41.6)
**Professional role *N* (%)**
Nurse	7 (29.2)
Nurse PC specialist	1 (4.2)
PC Physician	6 (25)
PC anesthetist	1 (4.2)
Psychologist	6 (25)
Physiotherapist	1 (4.2)
Spiritual support counselor	1 (4.2)

The study inclusion criteria were:

a) to be a healthcare professional or physician involved in a palliative care team;
b) to have worked in palliative care contexts in the regions most affected by the first wave of the virus (Lombardy, Piedmont, Veneto, and Tuscany).

The interviews were video-recorded and transcribed *verbatim*. The textual material was analyzed through the thematic analysis approach (Braun and Clarke, 2006). Following Braun and Clarke's six-phase framework, before starting coding, each member of the research team became familiar with the data reading independently through all the interview transcripts. Particularly, the interviews were analyzed by the research team to identify the full description of the participants' perspectives and experiences across all data. Initial codes were generated dividing the transcript texts into meaning units, describing and interpreting the most significant.

Then, they were selected and condensed into relevant themes, focused on the palliative role in COVID patient's management, especially in emergency and ICU departments:

- *Theme 1: The support of palliativists to doctors and health professionals;*
- *Theme 2: The experience of COVID for the reorganization of palliative and end-of-life care*.

Rigor and research validity were achieved by frequent discussing and sharing results in researchers' inter-analysis meetings and realizing an ordered and traceable series of cognitive actions (Rolfe, 2006).

Below are reported some results emerged from the interviews analysis, in particular in relation to the issue of end-of-life management in emergency contexts.

### The support of palliativists to doctors and health professionals

One of the critical issues that clearly emerged from the analysis of interviews concerned the support of palliative care specialists to doctors and health professionals in COVID wards. Their experience of support elicited several aspects. The first aspect concerns the management of the moment of death.

In many cases, the interviewees highlighted how their preparation for the management of the dying patient was more effective than other doctors and professionals deployed from other wards and even doctors from intensive care units. This aspect confirmed the specific expertise of the palliativist team, based on the logic of adaptation and resilience to death, and on the ability to balance personal and professional dimensions. These professional qualities unfortunately are often not a priority for medical professions who work in other areas and settings, as they are generally still anchored to the idea of resuscitation at all costs and the tendency of refusing the failure of medical interventions in end-of-life (McNamara et al., 1994).

“While I was working at the COVID High-Dependency ward, I realized the important contribution that palliative care can make in the emergency room and intensive care wards. This is because the colleagues from these areas who came to work with us on a team were immediately stressed especially when they saw patients die as happened during the first wave! We have had all these people coming in with severe dyspnea asking you ‘Help me! Help me!' They held tight to the sides of the bed and could not breathe! People died like this and these colleagues were so upset that they could not do anything to help them survive. Because all they knew was to resuscitate patients, while it was clear from the signs and symptoms, that the people who came to us were dying and that there was nothing we could do. In these situations, we (=the palliative care team) endeavored to provide sedation for the patients' unpleasant symptoms, while they (=the emergency team) have always tried to rescue these patients; but it was like wanting to bring patients back to life at any cost [...]. However, the collaboration between the two different teams has helped to understand how some conditions need less aggressive support and only an accompaniment to die. Moreover, we helped our colleagues in the emergency room to ascertain in which situations a more intense support is needed and in which it was important to let go instead”. (Nurse, int.16 - 2021)

Especially in the first wave, there were no guidelines and doctors operating in the emergency and COVID intensive care units had to change their approach quickly, often by trial and error. The burden of decisions and doubts about intubation or other resuscitation treatments have also given rise to psychological trauma for the professionals involved, not used to sharing their decisions within a multidisciplinary team.

“(…) But whether you think about the problem of intubating a patient or not. Having an expert there, for these decisions, (= a palliative care doctor) would clearly have made things faster and, how can I tell? There is that process of shared responsibility that makes the difference. However, I believe that resuscitators and anesthetists have no idea what this concept means. Therefore, they think these decisions are unnecessary (= shared end-of-life responsibility)”. (Psychologist, int.8 - 2020)

A second aspect concerns the communication with the dying patient. The literature has shown this issue still to be difficult to manage for doctors and healthcare professionals not accustomed to death and that, in some way, it still represents a problem in the management of end of life in intensive care (Glaser and Strauss, 1965; Bandini, 2020).

“I have always been used by them (= emergency ward) to report worsening patients' conditions to families. ‘I'm so lucky!' I told myself. However, while for me, it was very normal because I understood that it was a difficulty that I have overcome previously and that I have experienced this situation before. Therefore, during the emergency, the burden was on me and I had to take that responsibility (=in deployment)”. (PC Physician, int. 9 - 2020)

Finally, a third aspect, linked with specific skills of the palliative care team, consisted in the ability to relate to patients that were, for the emergency COVID situation, left alone because family and relatives weren't allowed to enter the wards. These patients had completely individual needs, depending on the physical state, age, disorders caused by COVID.

“[…]Dyspnea management is a situation that requires specific skills. It is an indescribable sensation that must be managed with the right medications and the right actions. And, from a relational point of view, being closer to the person, means establishing a relationship. It also means knowing how to ask and identify his needs, to support them to get in touch with other people. Knowing how to communicate and choose together what is most appropriate for them, showing what we can and cannot do. These are all skills that must be carried out by professionals who have undergone a specific learning path and who are basically palliativists”. (PC Physician, int.2 - 2020)

In some cases, palliativists have also managed to treat and heal some COVID patients in critical conditions, despite a prejudice against them by the intensive care staff, showing how this approach is not only able to alleviate suffering, but also to support treatment therapies (Masel and Kreye, 2018).

“Our goal was to go and provide palliative care. Instead, those who hosted us (=emergency COVID wards) expected us to somehow take away the workload, a job that would fail. Let's say that they could not spend energy on these sick people, because they were so sick and lost. Interesting, however, if one could reason with the statistics and if the period would have been a little longer, it could have emerged that the palliative care team, at some point, have cured and pulled out of the situation at least two or three people, who remain a little in the history now. No? They were able to give essential care to people who had somehow been abandoned therapeutically”. (Nurse PC Specialist, int.5 - 2020)

“Some dying patients admitted to hospice have come out of it (=COVID) simply because they have been treated effectively with palliative care. We have managed their shortness of breath, fever, cough, pain, and then all these aspects of the cure (=of COVID symptoms)”. (PC Physician, int.1 - 2020)

In a context of continuous and immediate deaths, lack of resources, exhausted and shocked personnel, the role of palliativists was the opposite of the principle of “social death” (Sudnow, 1967), even in those COVID patients who were already considered juts “bodies” by medical staff. Palliative care team tried to bring the treatments back to their “humanized” and personalized dimension, regardless of the patient's condition and the outcome of the therapeutic intervention.

“You cannot think of dealing with the end of life without having in mind the principles of ethics and bioethics. This implies that if you don't consider the human being, then you go back of being a technician. And, you will stop doing the palliative care professional at that point. To cut it short, it is essential. The identification of what the patient's choices are, what is their relational context, what is his/her idea of health, his/her idea of wellbeing, what are his/her decisions for their self-determination. You cannot ignore them. Otherwise, you would do another job. I would have applied to become an orthopedist. With all due respect to the orthopedic colleagues, right?” (PC Physician, int. 20 - 2021)

### The experience of COVID for the reorganization of palliative and end-of-life care

The COVID experience has certainly represented a challenge for the palliative culture and the management of the end of life even in emergency contexts (Fadul et al., 2021).

On this aspect, interviewees tried to reflect on the lessons learned and on some areas of intervention that can improve the management of the end of life in different healthcare contexts and situations. In particular, some have observed how palliative care should have undertaken before the patient's end of life and how both staff and patients in the emergency and intensive care wards could benefit from this.

“From this experience, it would have been right to untie anesthesiologists or operators in the critical area from having to assist people who would inevitably have died. I think this should have been done, precisely to avoid the extreme frustration that many of them have had in having to assist these people who, despite the care, attention, and everything, would not have made it. Palliative care teams were available from the start to care for these patients. Moreover, palliative care could have been recruited specifically to assist these patients, that is, these patients whose outcome was known to be unsuccessful”. (Physiotherapist, int.3 - 2020)

During the emergency there was substantially more action and less reflection on ethical, moral and practical implications of relevance of palliative approach also in the management COVID Patients.

“We did not have any studies that told us that if you intubate, some get along/through or not. What are the prognostic factors that tell you it will be good or bad? So it was all done in a hurry”. (Physician, int. 7, 2020)

“(…) there was no senior doctor, so they were junior anesthetists. (...) they didn't want to make decisions of a certain type. This was a problem. (...) The thing is (…) we treat people, and we do not treat objects so (...) you cannot leave certain responsibilities to trainees. (...) And, when in doubt I have always seen action and no stopping and trying to reflect for a moment”. (Nurse, int. 3, 2020)

On the other hand, thinking back to what they experienced during the critical phases of the pandemic, some interviewees highlighted the need to change the approach with respect to the “long time” of the end of life, mainly intended for dying patients.

“In my opinion, we had extraordinary expertise to spend. It is clear that it was a question of thinking about a new way of providing palliative care, that is, we could not think that it was always the same way. And, in my opinion, that was the interesting challenge, wasn't it? Because patients died even in two hours (=in COVID Wards). So, we didn't have the time for (=traditional) palliative care. However, it was a way to start thinking differently about end of life care. And, we missed this opportunity, so I really have this great regret”. (Psychologist, int. 8 - 2020)

Several interviewees reiterated that palliative care should be activated early in patients, to manage pain and suffering, despite the adverse prognosis outcome. This aspect is consistent with the palliative care scopes and aims, which are clearly not just for the last moments of the life of the person but that it must be an ongoing process from the diagnosis and before the insurgence of symptoms (Zagonel et al., 2017). Palliative care societies and associations are striving to support national health organizations to build a culture of palliation for long-term or chronic lethal diseases. In fact, most recent international guidelines suggest contemplating early the integration of palliative care in the path of anticancer treatments for all patients with advanced disease diagnoses (Armento et al., 2016). Therefore, palliative care could have an early activation and so act “simultaneously” with other treatments, health professionals, and care pathways that the person is undergoing, and this process can even last all life to maintain a balance and support the person with symptom control with a holistic perspective.

“It was understood that palliative care can act on several fronts, not only on the cancer patient, but also on other pathologies, and also on the territory. The family doctor recognized the value of palliative care in end-of-life care, and today we see palliative care activated for different patients than in the past, not just for oncology anymore”. (Nurse, int. 13 - 2021)

Lastly, some interviewees expressed their displeasure for having seen so many patients dying in solitude, without the presence of family and relatives around them. This principle goes against the palliative ethic, which considers the role of the family as an integral part of end-of-life care (Bakar et al., 2020).

“COVID has brought out the suffering of loneliness, isolation and lack of relationship, which are instead necessary to cure. People who died of COVID disease, who could not live the relationship and were left alone”. (Psychologist, int. 14 - 2021)

## Discussion

This work highlighted the complexity of end-of-life management and the need to rethink palliative care as a longitudinal competence integrated in various clinical-care areas, beyond the specific medical area (Julià-Torras et al., 2021).

The strategies highlighted by the sociological literature regarding the decision-making process of dying, as well as the practices for managing patient awareness or the buffet of options on advanced therapies to be shared with family members to accompany death, have proved in fact to be inapplicable during the acute phases of the pandemic. They showed how doctors, anesthetists and other professionals of intensive care or from other departments were all substantially unprepared to so many and unexpected patient's death in emergency conditions and with limited available health and clinical resources.

The management of so many COVID patients, often incurable or who have already entered the hospital in desperate conditions has indirectly shown how death still represents a conflicting and not yet resolved aspect of medicine, which marks a cultural and, in some cases, jurisdictional border between the expertise of the physician and that of the palliative care specialist. This expertise is based on the logic of adaptation and resilience to death and on the ability to balance personal and professional reflections (Singh et al., 2021).

The choice between keeping alive or letting die COVID patients has clashed with attempts, often useless, to recover patients who should have been only accompanied to death in a dignified, but also morally and ethically, acceptable way. In this situation, the role of palliative care specialists (when they were present) seemed to consist in providing not only the support for the management of pain and symptoms of COVID. They also showed to relate, in an individualized way, with dying patients, highlighting their needs, and, at the same time, to psychologically support doctors and health professionals, who were inevitably frustrated by having to continuously and rapidly manage so many deaths. The lack of interprofessional and shared decision-making practices among different health professionals and, particularly, palliative care professionals seems to have aggravated the “burden” of managing so many deaths during the critical phases of the pandemic and increased the sense of inadequacy in front of dying patients for many doctors and professionals not used to operating in emergency and terminal departments. This raises the question of greater reflection not only on the need to develop inter-professional teams also in emergency wards (Dreher-Hummel et al., 2021), but also on the relevance of palliative care experts in these sectors (Lamba et al., 2014).

In this study emerged how in emergency contexts, such as those that managed COVID patients during the acute phases of the pandemic, the action of healthcare professionals is often not accompanied by ethical reflection on what the initiated medical interventions implied (Falcó-Pegueroles et al., 2021). The speed of action and the desire to “do at any cost” is opposed to the assessment on the real needs of the dying patient and the necessity, as well as to heal, to bring medical personnel back to the acceptance of the inevitability of death. Consequently, the study also implicitly points out how the education and training on bioethical aspects and on palliative approaches is fundamental and how they could have supported many COVID staff in the decision-making process, avoiding the subsequent psychological traumas reported by many doctors and health professionals (Rosa et al., 2020; Donkers et al., 2021). What emerged by interviews is also a sort of “moral need” by palliative care professionals to educate e support their colleagues who work in other disciplines and especially in ICU and emergencies wards to manage end-of-life (Fadul et al., 2021; Hanna et al., 2021). This issue underlines the necessity to introduce end-of-life training and fundamental palliative care competencies into all the medical school and nursing curricula (Kim, 2020).

Therefore, this research aimed to point out that palliative care does not only manage patients' pain, but it can contribute significantly in supporting doctors and health professionals, from every healthcare setting, in dealing with different clinical and social situations emerging in terminal diseases scenarios. Besides, such scenarios represented all the palliative care spectrum of interventions, from the effective management of breathing and other symptoms to supporting doctors in choosing the “buffet” of options, without having to improperly delegate family members.

Nevertheless, the traditional biomedical vision of the holistic approach to palliative care as a medical failure (Bishop, 2011; Gawande, 2014) remains perhaps a significant aspect to reflect on, in order to overcome the medical perception of social death of dying patients and to give ethical relevance and quality of care to end-of life management. In our research, this cultural gap seems to be a critical point for the development of a more human, personalized and centered interprofessional approach on dying patients in healthcare settings such as emergencies and intensive care (McConnell et al., 2016).

## Conclusion

In conclusion, this brief analysis suggests the need to deepen and, probably, redefine the professional, ethical and clinical role of palliative care teams, not only in the management of COVID patients, but also in the healthcare system as a whole. This also requires a reflection on current clinical-care practices and on how to include an early integration of palliative care team knowledge and expertise at the service of collective and individual health (Zaborowski et al., 2022).

During the pandemic, perhaps looking at and facing the topic of death have certainly become everyone's business, but especially for doctors and health professionals. The palliative care model could help fill a gap in end-of-life care, which has persisted for years, by building a bridge between the ethics of care and the ethics of dying. Could this be one of the many lessons we could have learnt from the experience of this health emergency?

## Data availability statement

The datasets presented in this article are not readily available because they are part of a research that is in progress. Requests to access the datasets should be directed to barbara.sena@unitelmasapienza.it.

## Ethics statement

Ethical review and approval was not required for the study on human participants in accordance with the local legislation and institutional requirements. The patients/participants provided their written informed consent to participate in this study.

## Author contributions

All authors listed have made a substantial, direct, and intellectual contribution to the work and approved it for publication. However, for formal attribution reasons the Introduction, the paragraph on Sociological contributions on end of life care and the Discussion can be attributed to BS, the Results and the Conclusion to EDL.

## Conflict of interest

The authors declare that the research was conducted in the absence of any commercial or financial relationships that could be construed as a potential conflict of interest.

## Publisher's note

All claims expressed in this article are solely those of the authors and do not necessarily represent those of their affiliated organizations, or those of the publisher, the editors and the reviewers. Any product that may be evaluated in this article, or claim that may be made by its manufacturer, is not guaranteed or endorsed by the publisher.
